# Supplementary material for: EnzML: multi-label prediction of enzyme classes using InterPro signatures
Source: BMC Bioinformatics. 2012 Apr 25;13:61. doi: 10.1186/1471-2105-13-61 (PMC3483700; doi:10.1186/1471-2105-13-61)
Supplement: Addtional file 5 — The Java code to format the data files, evaluate and predict. The file enzml_java_code.tar.gz contains the Java code used to format database data to ARFF and XML formats, to execute cross and train-test (jackknife) evaluations and to record evaluation results to database. More information is included in the readme.txt file and the Javadoc files. The code can be used with a MySQL database. To use a different database software, other JDBC drivers might be required. [file 1471-2105-13-61-S5.gz › java_code/enzml2011/doc/uk/ac/ed/inf/taweka/mulan/MulanArffProperties.html]

MulanArffProperties


---


|  |  |  |
| --- | --- | --- |
| **Overview** \| **Package** \| **Class** \| **Use** \| **Tree** \| **Deprecated** \| **Index** \| **Help** | |  |
| **PREV CLASS**   **NEXT CLASS** | **FRAMES**    **NO FRAMES**     **All Classes** |  |
| SUMMARY: NESTED \| FIELD \| CONSTR \| METHOD | DETAIL: FIELD \| CONSTR \| METHOD |  |


---


## uk.ac.ed.inf.taweka.mulan Class MulanArffProperties

```
java.lang.Object
  uk.ac.ed.inf.taweka.Initialised
      uk.ac.ed.inf.taweka.weka.ArffProperties
          uk.ac.ed.inf.taweka.mulan.MulanArffProperties
```

**All Implemented Interfaces:**: Initialisable

---

``` public class MulanArffProperties extends ArffProperties ```

Class

**Version:**
:   14 Sep 2010

**Author:**
:   Luna De Ferrari luna.deferrari-at-ed.ac.uk

---

| **Field Summary** | |
| --- | --- |
| `static java.lang.String` | `XML_QUERY_PROP`             The query to get the class labels for the Mulan xml file (if needed) |

**Fields inherited from class uk.ac.ed.inf.taweka.weka.ArffProperties**

`ARFF_FILE_PATH_PROP, CLASS_NAME_PROP, DATA_PROPERTIES_FILE_PATH_PROP, DATASET_NAME_PROP, DESCRIPTION_PROP, INSTANCE_ATTRIBUTE_QUERY_PROP, INSTANCE_CLASS_QUERY_PROP, NUMBER_OF_ATTRIBUTES, NUMBER_OF_CLASS_VALUES, NUMBER_OF_CORE_PROPERTIES, NUMBER_OF_INSTANCES, NUMBER_OF_STATISTICS_PROPERTIES, TIMESTAMP_FIELD`

**Fields inherited from class uk.ac.ed.inf.taweka.Initialised**

`m_initialisationIsCorrect`


**Constructor Summary**

`MulanArffProperties(java.lang.String propertiesFilePath)`


| **Method Summary** | |
| --- | --- |
| `java.util.TreeSet<java.lang.String>` | `propertiesKeys()`             All properties: core + statistics |

**Methods inherited from class uk.ac.ed.inf.taweka.weka.ArffProperties**

`checkPropertiesKeys, coreKeys, corePropertiesAreCorrect, getArffFilePath, getDbConn, getProperties, getPropertiesFilePath, getProperty, propertiesAreCorrect, saveStatisticsAsProperties, statisticsKeys, statsPropertiesAreCorrect, toString`

**Methods inherited from class uk.ac.ed.inf.taweka.Initialised**

`initialisationIsCorrect, setInitialisationIsCorrect`

**Methods inherited from class java.lang.Object**

`equals, getClass, hashCode, notify, notifyAll, wait, wait, wait`

**Field Detail**

### XML\_QUERY\_PROP

```
public static final java.lang.String XML_QUERY_PROP
```

:   The query to get the class labels for the Mulan xml file (if needed)

    **See Also:**: Constant Field Values


**Constructor Detail**

### MulanArffProperties

```
public MulanArffProperties(java.lang.String propertiesFilePath)
```


**Method Detail**

### propertiesKeys

```
public java.util.TreeSet<java.lang.String> propertiesKeys()
```

:   **Description copied from class: `ArffProperties`**
:   All properties: core + statistics

    :   **Overrides:**: `propertiesKeys` in class `ArffProperties`
    :   **Returns:**


---


|  |  |  |
| --- | --- | --- |
| **Overview** \| **Package** \| **Class** \| **Use** \| **Tree** \| **Deprecated** \| **Index** \| **Help** | |  |
| **PREV CLASS**   **NEXT CLASS** | **FRAMES**    **NO FRAMES**     **All Classes** |  |
| SUMMARY: NESTED \| FIELD \| CONSTR \| METHOD | DETAIL: FIELD \| CONSTR \| METHOD |  |


---
